# Crystal structure of di­aqua­tris­(benzohydrazide-κ^2^ *N*,*O*)(isophthalato-κ*O*)samarium(III) nitrate

**DOI:** 10.1107/S2056989018015360

**Published:** 2018-11-06

**Authors:** Chatphorn Theppitak, Filip Kielar, Kittipong Chainok

**Affiliations:** a Division of Chemistry, Faculty of Science and Technology, Thammasat University, Khlong Luang, Pathum Thani, 12121, Thailand; b Department of Chemistry, Faculty of Science, Naresuan University, Muang, Phitsanulok, 65000, Thailand; c Materials and Textile Technology, Faculty of Science and Technology, Thammasat University, Khlong Luang, Pathum Thani, 12121, Thailand

**Keywords:** crystal structure, hydrogen bonds, lanthanide, samarium(III)

## Abstract

The first benzohydrazide complex of a lanthanide is reported. In the title compound, [Sm(C_8_H_4_O_4_)(C_7_H_8_N_2_O)_3_(H_2_O)_2_]NO_3_, systematic name di­aqua­tris­(benzohydrazide-κ^2^ *N*,*O*)(isophthalato-κ*O*)samarium(III) nitrate, the Sm^III^ ion is nine-coordinated in a distorted tricapped trigonal–prismatic geometry by three oxygen atoms and three nitro­gen atoms from three benzhydrazide (bzz) ligands, one oxygen atom from the isophthalate (itp^2−^) ligand, and two oxygen atoms from coordinated water mol­ecules. The nitrate group is disordered over two sets of sites with occupancy factors of 0.310 (17) and 0.690 (17). In the crystal, adjacent mol­ecules are linked into chains *via* pairs of O—H⋯O and N—H⋯O hydrogen bonds between the carboxyl­ate acceptor and the coordinated water and amine NH_2_ donors. Mol­ecules are further stacked by π–π inter­actions involving the benzene ring of the itp^2−^ ligands, forming double chains that extend in the *b*-axis direction. These double chains are further linked into a three-dimensional supra­molecular network *via* hydrogen bonds (O—H⋯O, N—H⋯O and C—H⋯O) between the complex mol­ecule and the nitrate groups along with C—H⋯π and π–π inter­actions involving the benzene rings of the bzz and itp^2−^ ligands.

## Abstract 2

The first benzohydrazide complex of a lanthanide is reported. The Sm^III^ ion is nine-coordinated in a distorted tricapped trigonal–prismatic geometry by three oxygen atoms and three nitro­gen atoms from three benzhydrazide (bzz) ligands, one oxygen atom from the isophthalate (itp^2−^) ligand, and two oxygen atoms from coordinated water mol­ecules. The crystal structure features extensive hydrogen bonding as well as C—H⋯π and π–π inter­actions.

## Chemical context   

Research on lanthanide-based coordination compounds is one of the most active fields in chemistry and materials science. Distinct from transition metal centers, lanthanide ions often demonstrate high and variable coordination numbers as well as diverse coordination geometries, which can lead to versatile structures and topologies (Cotton & Raithby, 2017 ▸). They are also very attractive luminescent centers for the high colour purity and relatively long lifetimes arising from electronic transitions within the partially filled 4*f* orbitals, which make them potential candidates for applications in lighting, photon­ics and as luminescent probes and sensors (Parker, 2000 ▸; Bünzli & Piguet, 2005 ▸; Cui *et al.*, 2018 ▸). Besides the metal ions, the organic ligands also have significant effects on the construction of novel lanthanide coordination compounds and their potential applications (Lu *et al.*, 2012 ▸; Xu *et al.*, 2016 ▸; You *et al.*, 2018 ▸). It is well-known that lanthanide ions have a high affinity for and prefer to bind to hard donor atoms such as oxygen-containing organic ligands, for instance aromatic carb­oxy­lic acids. Terephthalic acid and its derivatives have thus been widely employed in the synthesis of novel lanthanide-based coordination compounds with inter­esting architectures and photoluminescence properties (Karmakar *et al.*, 2016 ▸; Park & Oh, 2016 ▸). These ligands can exhibit various coordination modes when coordinated to the metal centers, as well as serving as antennas or sensitizers to absorb light and transfer energy to the excited states of the central lanthanide ions (Bünzli & Piguet, 2005 ▸). Aromatic organic compounds containing the hydrazide group have been used widely as chemical receptors for sensing anions (Ran *et al.*, 2017 ▸; Liu *et al.*, 2018 ▸), but have received less attention as metal chelators. In a search for new structural chemistry, we employed benzhydrazide (bzz) and isoterephthalic acid (H_2_itp) as ligands to react with Sm(NO_3_)_3_·6H_2_O under hydro­thermal conditions, and the crystal structure determination of the title compound is reported herein.
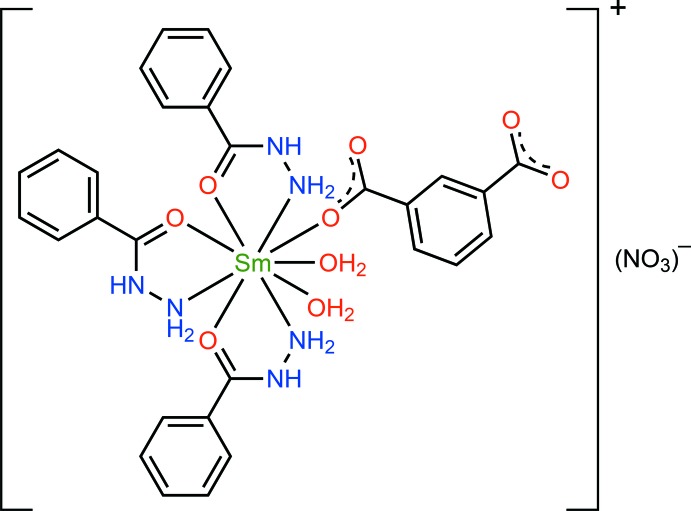


## Structural commentary   

**Figure 1:**
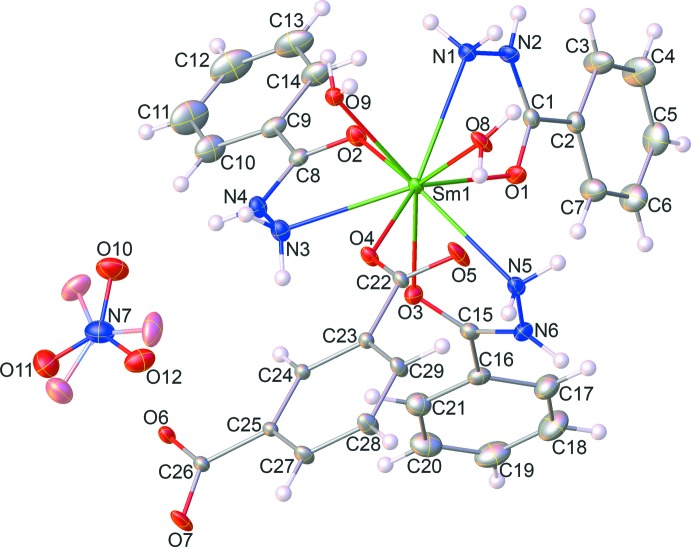
Mol­ecular structure of the title compound, showing the atom-labelling scheme. Displacement ellipsoids are drawn at the 30% probability level.

**Figure 2:**
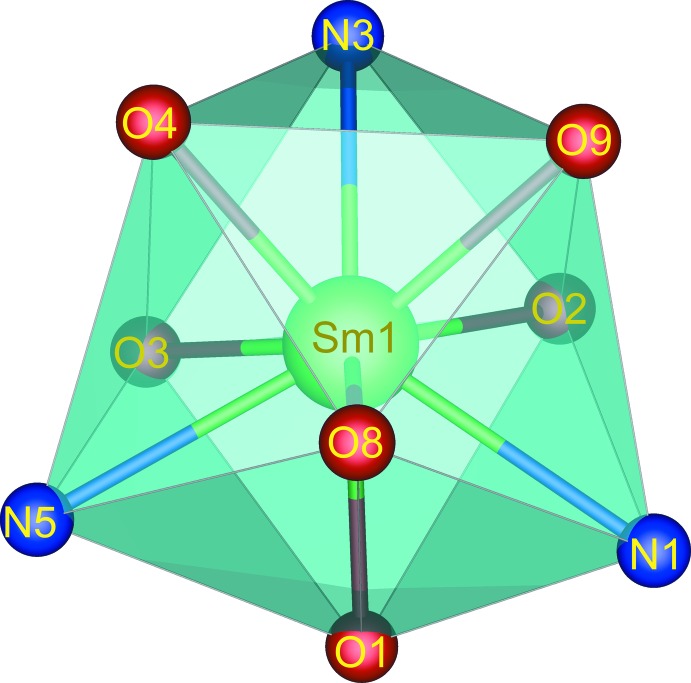
View of the distorted tricapped trigonal–prismatic coordination geometry of the central Sm^III^ atom.

The mol­ecular structure of the title compound is shown in Fig. 1 ▸. The asymmetric unit comprises one Sm^III^ ion, three benzhydrazide (bzz) ligands, one completely deprotonated isophthalate (itp^2−^) ligand, two coordinated water mol­ecules, and one disordered NO_3_^−^ ion. The hydrazide group of the bzz ligand adopts a bidentate *μ*_2_-η^1^:η^1^ chelating coordination mode, whereas the carboxyl­ate groups of the fully deprotonated itp^2−^ ligand display a *μ*_1_-η^1^:η^0^ monodentate coordination fashion. The Sm^III^ ion is nine-coordinated by three oxygen atoms (O1, O2, O3) and three nitro­gen atoms (N1, N3, N5) of three different bzz ligands, one oxygen atom (O4) from the completely deprotonated itp^2−^ ligand, and other two oxygen atoms (O8, O9) from the coordinated water mol­ecules. The central metal Sm^III^ atom can be described as having a distorted tricapped trigonal–prismatic geometry, Fig. 2 ▸, with the Sm—N and the Sm—O bond lengths in the ligand ranging from 2.633 (2) to 2.694 (2) Å and 2.340 (2) to 2.478 (2) Å, respectively, and the N/O—Sm—N/O bond angles fall in the range 60.97 (6) to 145.24 (6)°. These values are comparable to other reported values for oxygen/nitro­gen-coordinated Sm^III^ complexes (Alipour *et al.*, 2016 ▸; An *et al.*, 2016 ▸).

## Supra­molecular features   

**Figure 3:**
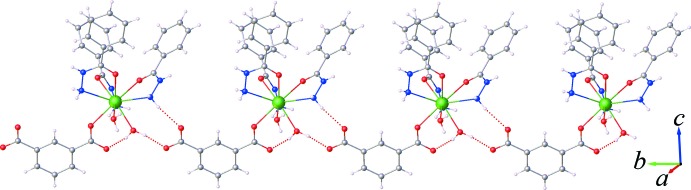
View of a supra­molecular chain formed by O—H⋯O and N—H⋯O hydrogen bonds.

**Figure 4:**
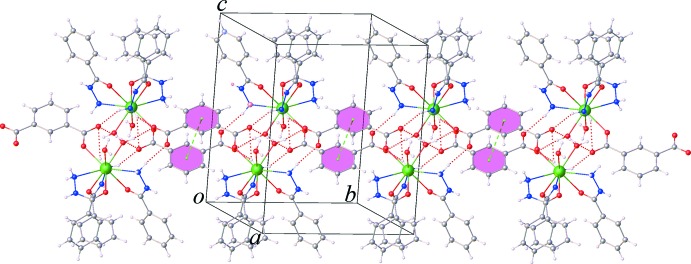
View of a supra­molecular double chain sustained by O—H⋯O and N—H⋯O hydrogen bonding along with π–π stacking inter­actions.

**Figure 5:**
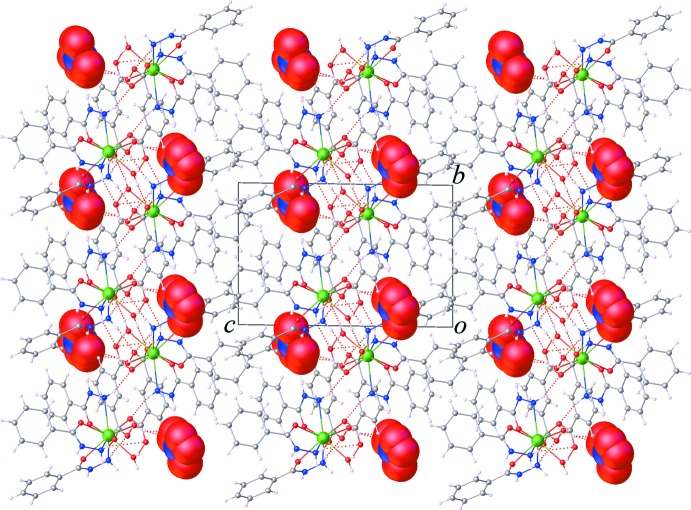
The crystal packing of the title compound, viewed along the *a* axis. The nitrate mol­ecules are shown with a space-filling model.

As can be seen in Fig. 3 ▸, one carboxyl­ate group of the itp^2−^ ligand adopts a monodentate coordination mode to the Sm^III^ ion, while the other acts as an acceptor of hydrogen-bonding inter­actions (Table 1 ▸) involving the water mol­ecules and the amine NH_2_ group of the bzz ligand of an adjacent complex mol­ecules. This arrangement gives rise to chains extending along the *b*-axis direction by offset π–π inter­actions between the benzene rings of symmetry-related itp^2−^ ligands, Fig. 4 ▸, with a centroid to centroid distance of *Cg*4⋯*Cg*4^i^ = 3.692 (2) Å and a dihedral angle = 0.0 (2)° [*Cg*4 is the centroid of the C23–C29 ring; symmetry code: (i) 2 − *x*, 1 − *y*, 1 − *z*). Fig. 5 ▸ shows the crystal packing of the title compound along the *a* axis. The three-dimensional supra­mol­ecular architecture of the crystal is sustained by numerous O—H⋯O, N—H⋯O and C—H⋯O hydrogen bonds between the complex mol­ecules and the nitrate groups along with weak C—H⋯π inter­actions between the aromatic C—H bonds and the benzene rings of the bzz ligands, Table 1 ▸. Furthermore, weak aromatic π–π stacking inter­actions involving the bzz ligands [*Cg*1⋯*Cg*1^ii^ = 3.882 (2) Å, dihedral angle = 0.0 (5)°; *Cg*1 is the centroid of the C2–C7 ring; symmetry code: (ii) 1 − *x*, −*y*, 2 − *z*;] , and the bzz and itp^2−^ ligands [*Cg*2⋯*Cg*4^iii^ = 3.715 (3) Å, dihedral angle = 4.7 (9)°; *Cg*2 is the centroid of the C9–C14 ring; symmetry code: (iii) 1 − *x*, 1 − *y*, 1 − *z*], are also observed, which help further to stabilize the crystal structure.

## Database survey   

A search of the Cambridge Structural Database (CSD, version 5.39, last update August 2018; Groom *et al.*, 2016 ▸) gave 20 hits for the benzohydrazide complexes with transition metal ions, but none of them involves a lanthanide ion. The most typical coordination mode of benzohydrazide ligands in structures appears to be a bidentate chelating mode with metal centers through nitro­gen and oxygen donor atoms (BOHYCU, Nyburg *et al.*, 1971 ▸; EKAMIM, Odunola *et al.*, 2003 ▸; EZARED, EZARIH, Patel *et al.*, 2011 ▸; XUQYUD01, Thiam *et al.*, 2009 ▸). In these complexes, the nitro­gen atoms of the hydrazide group serve as donors for hydrogen bonding.

## Synthesis and crystallization   

A mixture of Sm(NO_3_)_3_·6H_2_O (44.5 mg, 0.1 mmol), bzz (27.4 mg, 0.2 mmol), H_2_itp (16.5 mg, 0.1 mmol), and H_2_O (4 ml) was sealed in a 15 ml Teflon-lined steel autoclave and heated at 373 K for 24 h. The mixture was cooled to room temperature and light-yellow block-shaped crystals of the title compound were obtained in 79% yield (35.2 mg, based on Sm^III^ source). Analysis calculated (%) for C_29_H_32_N_7_O_12_Sm (1376.80): C 42.43; H 3.93; N 11.94%. Found: C 42.46; H 3.96; N 11.90%.

## Refinement   

Crystal data, data collection and structure refinement details are summarized in Table 2 ▸. All H atoms were located in difference maps. The H atoms bonded to C atoms were treated as riding atoms in geometrically idealized position with C—H distances of 0.93 Å and with *U*_iso_(H) = 1.2*U*_eq_(C). The H atoms bonded to O and N atoms were located in a difference-Fourier map, but were refined with distance restraints of O—H = 0.84 ± 0.01 Å and N—H = 0.88 ± 0.01 Å, and with *U*_iso_(H) = 1.5*U*_eq_(O) and 1.2*U*_eq_(N). The nitrate group is disordered over two sets of sites, with occupancy factors of 0.310 (17) and 0.690 (17).

## Supplementary Material

Crystal structure: contains datablock(s) I. DOI: 10.1107/S2056989018015360/pj2059sup1.cif

Structure factors: contains datablock(s) I. DOI: 10.1107/S2056989018015360/pj2059Isup2.hkl

Supporting information file. DOI: 10.1107/S2056989018015360/pj2059Isup3.cdx

CCDC reference: 1876239

Additional supporting information:  crystallographic information; 3D view; checkCIF report

## Figures and Tables

**Table 1:** Hydrogen-bond geometry (Å, °) *Cg*1 and *Cg*3 are the centroids of the C2–C7 and C16–C21 rings, respectively.

*D*—H⋯*A*	*D*—H	H⋯*A*	*D*⋯*A*	*D*—H⋯*A*
O8—H8*A*⋯O7^i^	0.83 (2)	1.75 (2)	2.574 (2)	168 (3)
O8—H8*B*⋯O5	0.84 (2)	1.76 (2)	2.585 (3)	166 (3)
O9—H9*A*⋯O12*A* ^ii^	0.83 (2)	2.22 (2)	2.988 (2)	156 (3)
O9—H9*A*⋯O12*B* ^ii^	0.83 (2)	2.09 (2)	2.888 (2)	162 (3)
O9—H9*B*⋯O6^ii^	0.83 (2)	1.81 (2)	2.633 (2)	170 (3)
N1—H1*A*⋯O6^i^	0.88 (2)	2.14 (2)	3.012 (3)	172 (2)
N1—H1*B*⋯O6^ii^	0.88 (2)	2.14 (2)	2.965 (3)	156 (3)
N2—H2⋯O11*A* ^i^	0.87 (2)	2.22 (3)	2.936 (2)	140 (2)
N2—H2⋯O12*B* ^i^	0.87 (2)	2.10 (2)	2.946 (3)	164 (3)
N3—H3*B*⋯O4^ii^	0.88 (2)	2.53 (2)	3.344 (3)	155 (3)
N4—H4⋯O10*B*	0.87 (2)	2.18 (2)	3.040 (3)	176 (3)
N4—H4⋯O12*A*	0.87 (2)	2.31 (3)	2.970 (2)	133 (3)
N5—H5*A*⋯O5^iii^	0.88 (2)	2.07 (2)	2.878 (3)	152 (2)
N5—H5*B*⋯O5	0.87 (2)	2.67 (2)	3.199 (3)	120 (2)
N5—H5*B*⋯O7^iv^	0.87 (2)	2.15 (2)	2.940 (3)	151 (2)
N6—H6⋯O10*A* ^v^	0.87 (2)	2.22 (3)	2.961 (2)	142 (2)
N6—H6⋯O11*B* ^v^	0.87 (2)	2.28 (2)	3.115 (3)	159 (3)
C10—H10⋯O10*B*	0.93	2.36	3.282 (2)	173
C11—H11⋯*Cg*1^vi^	0.93	3.10	3.866 (2)	137
C13—H13⋯*Cg*3^vii^	0.93	3.02	3.712 (4)	132

Symmetry codes: (i) 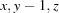; (ii) 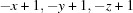; (iii) 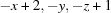; (iv) 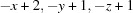; (v) 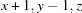; (vi) 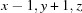; (vii) 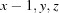.

**Table 2:** Experimental details

Crystal data	
Chemical formula	[Sm(C_8_H_4_O_4_)(C_7_H_8_N_2_O)_3_(H_2_O)_2_]NO_3_
*M*_r_	820.96
Crystal system, space group	Triclinic, *P* 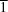
Temperature (K)	296
*a*, *b*, *c* (Å)	11.0784 (12), 11.2518 (13), 15.3590 (18)
α, β, γ (°)	84.039 (4), 78.487 (4), 62.042 (3)
*V* (Å^3^)	1656.9 (3)
*Z*	2
Radiation type	Mo *K*α
μ (mm^−1^)	1.84
Crystal size (mm)	0.22 × 0.2 × 0.2

Data collection	
Diffractometer	Bruker D8 QUEST CMOS
Absorption correction	Multi-scan (*SADABS*; Bruker, 2016 ▸)
*T*_min_, *T*_max_	0.698, 0.746
No. of measured, independent and observed [*I* > 2σ(*I*)] reflections	52341, 7637, 6577
*R*_int_	0.056
(sin θ/λ)_max_ (Å^−1^)	0.652

Refinement	
*R*[*F* ^2^ > 2σ(*F* ^2^)], *wR*(*F* ^2^), *S*	0.027, 0.053, 1.05
No. of reflections	7637
No. of parameters	531
No. of restraints	55
H-atom treatment	H atoms treated by a mixture of independent and constrained refinement
Δρ_max_, Δρ_min_ (e Å^−3^)	0.47, −0.36

Computer programs: *APEX3* and *SAINT* (Bruker, 2016 ▸), *SHELXT* (Sheldrick, 2015*a* ▸), *SHELXL2014* (Sheldrick, 2015*b* ▸) and *OLEX2* (Dolomanov *et al.*, 2009 ▸).

## supplementary crystallographic information

### Crystal data

**Table:**

[Sm(C_8_H_4_O_4_)(C_7_H_8_N_2_O)_3_(H_2_O)_2_]NO_3_	*Z* = 2
*M**_r_* = 820.96	*F*(000) = 826
Triclinic, *P*1	*D*_x_ = 1.645 Mg m^−^^3^
*a* = 11.0784 (12) Å	Mo *K*α radiation, λ = 0.71073 Å
*b* = 11.2518 (13) Å	Cell parameters from 9800 reflections
*c* = 15.3590 (18) Å	θ = 3.1–27.3°
α = 84.039 (4)°	µ = 1.84 mm^−^^1^
β = 78.487 (4)°	*T* = 296 K
γ = 62.042 (3)°	Block, light yellow
*V* = 1656.9 (3) Å^3^	0.22 × 0.2 × 0.2 mm

### Data collection

**Table:**

Bruker D8 QUEST CMOS diffractometer	7637 independent reflections
Radiation source: microfocus sealed x-ray tube, Incoatec Iµus	6577 reflections with *I* > 2σ(*I*)
GraphiteDouble Bounce Multilayer Mirror monochromator	*R*_int_ = 0.056
Detector resolution: 10.5 pixels mm^-1^	θ_max_ = 27.6°, θ_min_ = 3.1°
φ and ω scans	*h* = −14→14
Absorption correction: multi-scan (SADABS; Bruker, 2016)	*k* = −14→14
*T*_min_ = 0.698, *T*_max_ = 0.746	*l* = −19→20
52341 measured reflections	

### Refinement

**Table:**

Refinement on *F*^2^	Primary atom site location: dual
Least-squares matrix: full	Hydrogen site location: mixed
*R*[*F*^2^ > 2σ(*F*^2^)] = 0.027	H atoms treated by a mixture of independent and constrained refinement
*wR*(*F*^2^) = 0.053	*w* = 1/[σ^2^(*F*_o_^2^) + (0.0209*P*)^2^ + 0.7036*P*] where *P* = (*F*_o_^2^ + 2*F*_c_^2^)/3
*S* = 1.05	(Δ/σ)_max_ = 0.001
7637 reflections	Δρ_max_ = 0.47 e Å^−^^3^
531 parameters	Δρ_min_ = −0.36 e Å^−^^3^
55 restraints	

### Special details

**Table:**

Geometry. All esds (except the esd in the dihedral angle between two l.s. planes) are estimated using the full covariance matrix. The cell esds are taken into account individually in the estimation of esds in distances, angles and torsion angles; correlations between esds in cell parameters are only used when they are defined by crystal symmetry. An approximate (isotropic) treatment of cell esds is used for estimating esds involving l.s. planes.

### Fractional atomic coordinates and isotropic or equivalent isotropic displacement parameters (Å^2^)

**Table:**

	*x*	*y*	*z*	*U*_iso_*/*U*_eq_	Occ. (<1)
Sm1	0.64247 (2)	0.20819 (2)	0.60370 (2)	0.02461 (4)	
O1	0.68088 (19)	0.03820 (18)	0.72193 (12)	0.0396 (4)	
O2	0.41572 (18)	0.28637 (17)	0.69227 (12)	0.0384 (4)	
O3	0.67732 (18)	0.29498 (17)	0.73343 (12)	0.0368 (4)	
O4	0.74767 (17)	0.32614 (15)	0.50425 (11)	0.0320 (4)	
O5	0.93740 (19)	0.16420 (17)	0.43030 (15)	0.0507 (5)	
O6	0.69780 (16)	0.79297 (15)	0.48404 (12)	0.0337 (4)	
O7	0.88238 (19)	0.81877 (17)	0.42352 (14)	0.0466 (5)	
O8	0.7757 (2)	0.05576 (18)	0.48831 (14)	0.0409 (5)	
H8A	0.799 (3)	−0.0206 (17)	0.470 (2)	0.069 (11)*	
H8B	0.836 (3)	0.079 (3)	0.465 (2)	0.075 (12)*	
O9	0.50028 (18)	0.28060 (17)	0.48404 (12)	0.0316 (4)	
H9A	0.548 (2)	0.243 (3)	0.4371 (11)	0.036 (8)*	
H9B	0.431 (2)	0.267 (3)	0.493 (2)	0.055 (10)*	
O10A	0.1374 (12)	0.878 (2)	0.7680 (9)	0.066 (5)	0.310 (17)
O10B	0.1940 (8)	0.8003 (8)	0.7345 (4)	0.0735 (19)	0.690 (17)
O11A	0.266 (2)	0.9732 (16)	0.7463 (14)	0.088 (5)	0.310 (17)
O11B	0.2010 (9)	0.9791 (7)	0.7585 (5)	0.079 (2)	0.690 (17)
O12A	0.357 (2)	0.7680 (15)	0.7052 (12)	0.076 (5)	0.310 (17)
O12B	0.3861 (6)	0.8098 (8)	0.6970 (5)	0.0613 (16)	0.690 (17)
N1	0.5415 (2)	0.0317 (2)	0.60532 (14)	0.0312 (5)	
H1A	0.589 (2)	−0.0320 (19)	0.5659 (13)	0.036 (8)*	
H1B	0.4578 (15)	0.074 (3)	0.5919 (19)	0.048 (9)*	
N2	0.5377 (2)	−0.0322 (2)	0.68934 (14)	0.0347 (5)	
H2	0.487 (2)	−0.073 (2)	0.7027 (18)	0.042 (8)*	
N3	0.4835 (2)	0.4741 (2)	0.62389 (15)	0.0358 (5)	
H3A	0.534 (3)	0.503 (3)	0.6428 (18)	0.047 (9)*	
H3B	0.448 (3)	0.520 (3)	0.5775 (14)	0.062 (10)*	
N4	0.3651 (2)	0.5034 (2)	0.68984 (14)	0.0331 (5)	
H4	0.313 (3)	0.5874 (13)	0.7034 (19)	0.050 (9)*	
N5	0.8956 (2)	0.1034 (2)	0.63928 (15)	0.0318 (5)	
H5A	0.930 (3)	0.0158 (11)	0.6346 (18)	0.040 (8)*	
H5B	0.949 (2)	0.131 (3)	0.6035 (15)	0.037 (8)*	
N6	0.8946 (2)	0.1288 (2)	0.72747 (15)	0.0356 (5)	
H6	0.9703 (18)	0.084 (2)	0.7502 (17)	0.044 (8)*	
N7A	0.2522 (11)	0.8721 (11)	0.7372 (13)	0.032 (4)	0.310 (17)
N7B	0.2615 (8)	0.8629 (9)	0.7303 (8)	0.056 (2)	0.690 (17)
C1	0.6114 (3)	−0.0239 (2)	0.74407 (17)	0.0325 (6)	
C2	0.6105 (3)	−0.0903 (3)	0.83308 (18)	0.0368 (6)	
C3	0.5432 (4)	−0.1672 (4)	0.8608 (2)	0.0667 (10)	
H3	0.495609	−0.181394	0.822857	0.080*	
C4	0.5465 (5)	−0.2234 (4)	0.9454 (3)	0.0846 (13)	
H4A	0.500847	−0.275456	0.963900	0.101*	
C5	0.6148 (4)	−0.2039 (4)	1.0014 (2)	0.0693 (10)	
H5	0.616239	−0.242175	1.058118	0.083*	
C6	0.6809 (4)	−0.1287 (4)	0.9749 (2)	0.0681 (10)	
H6A	0.728290	−0.115280	1.013370	0.082*	
C7	0.6789 (4)	−0.0715 (3)	0.8912 (2)	0.0563 (8)	
H7	0.724585	−0.019298	0.873923	0.068*	
C8	0.3369 (2)	0.4028 (3)	0.71862 (16)	0.0310 (5)	
C9	0.2075 (3)	0.4315 (3)	0.78336 (17)	0.0409 (7)	
C10	0.1151 (3)	0.5588 (4)	0.8150 (2)	0.0631 (9)	
H10	0.133562	0.630937	0.796580	0.076*	
C11	−0.0048 (4)	0.5792 (6)	0.8740 (3)	0.0937 (15)	
H11	−0.066925	0.665074	0.895052	0.112*	
C12	−0.0318 (5)	0.4751 (7)	0.9009 (3)	0.0989 (17)	
H12	−0.112158	0.489572	0.941059	0.119*	
C13	0.0568 (4)	0.3492 (6)	0.8701 (3)	0.0827 (13)	
H13	0.036416	0.278359	0.888977	0.099*	
C14	0.1782 (3)	0.3254 (4)	0.8102 (2)	0.0588 (9)	
H14	0.238632	0.239352	0.788698	0.071*	
C15	0.7799 (3)	0.2250 (2)	0.77007 (17)	0.0335 (6)	
C16	0.7760 (3)	0.2465 (3)	0.86489 (18)	0.0394 (6)	
C17	0.8465 (4)	0.1409 (4)	0.9196 (2)	0.0587 (9)	
H17	0.901844	0.054955	0.896659	0.070*	
C18	0.8347 (4)	0.1631 (5)	1.0086 (3)	0.0787 (12)	
H18	0.881011	0.091693	1.045469	0.094*	
C19	0.7552 (4)	0.2898 (5)	1.0424 (3)	0.0776 (12)	
H19	0.748237	0.304574	1.102061	0.093*	
C20	0.6865 (4)	0.3939 (4)	0.9889 (2)	0.0665 (10)	
H20	0.633608	0.480011	1.012134	0.080*	
C21	0.6941 (3)	0.3734 (3)	0.9001 (2)	0.0529 (8)	
H21	0.644298	0.444845	0.864386	0.064*	
C22	0.8656 (2)	0.2830 (2)	0.45328 (16)	0.0280 (5)	
C23	0.9215 (2)	0.3801 (2)	0.42054 (15)	0.0243 (5)	
C24	0.8477 (2)	0.5137 (2)	0.44522 (15)	0.0233 (5)	
H24	0.761853	0.543431	0.482257	0.028*	
C25	0.9000 (2)	0.6041 (2)	0.41547 (15)	0.0239 (5)	
C26	0.8210 (2)	0.7487 (2)	0.44296 (16)	0.0275 (5)	
C27	1.0285 (3)	0.5579 (2)	0.36103 (17)	0.0336 (6)	
H27	1.064915	0.617294	0.341062	0.040*	
C28	1.1032 (3)	0.4249 (3)	0.33605 (19)	0.0398 (6)	
H28	1.189294	0.395113	0.299318	0.048*	
C29	1.0498 (2)	0.3361 (2)	0.36574 (17)	0.0331 (6)	
H29	1.100071	0.246465	0.348907	0.040*	

### Atomic displacement parameters (Å^2^)

**Table:**

	*U*^11^	*U*^22^	*U*^33^	*U*^12^	*U*^13^	*U*^23^
Sm1	0.02326 (7)	0.01824 (6)	0.03323 (7)	−0.01103 (5)	−0.00329 (5)	0.00044 (4)
O1	0.0417 (10)	0.0396 (10)	0.0507 (11)	−0.0292 (9)	−0.0171 (9)	0.0155 (9)
O2	0.0333 (10)	0.0308 (10)	0.0500 (11)	−0.0170 (8)	0.0014 (8)	−0.0011 (8)
O3	0.0314 (10)	0.0306 (9)	0.0443 (11)	−0.0085 (8)	−0.0096 (8)	−0.0066 (8)
O4	0.0293 (9)	0.0208 (8)	0.0441 (10)	−0.0136 (7)	0.0044 (8)	−0.0035 (7)
O5	0.0391 (11)	0.0220 (9)	0.0847 (15)	−0.0165 (8)	0.0179 (10)	−0.0171 (9)
O6	0.0241 (9)	0.0201 (8)	0.0562 (11)	−0.0096 (7)	−0.0036 (8)	−0.0069 (8)
O7	0.0390 (10)	0.0245 (9)	0.0792 (14)	−0.0221 (8)	0.0088 (10)	−0.0119 (9)
O8	0.0371 (10)	0.0243 (10)	0.0609 (13)	−0.0182 (9)	0.0106 (9)	−0.0149 (9)
O9	0.0272 (10)	0.0291 (9)	0.0395 (11)	−0.0144 (8)	−0.0041 (8)	−0.0002 (8)
O10A	0.034 (5)	0.093 (11)	0.072 (7)	−0.031 (6)	−0.010 (4)	0.008 (7)
O10B	0.058 (3)	0.078 (4)	0.100 (4)	−0.048 (3)	0.002 (3)	−0.017 (3)
O11A	0.071 (10)	0.050 (6)	0.152 (12)	−0.030 (7)	−0.025 (9)	−0.011 (6)
O11B	0.079 (5)	0.052 (3)	0.103 (4)	−0.020 (3)	−0.028 (4)	−0.016 (2)
O12A	0.058 (8)	0.051 (7)	0.078 (7)	0.014 (5)	−0.016 (6)	−0.020 (6)
O12B	0.047 (2)	0.084 (4)	0.058 (3)	−0.034 (2)	−0.0040 (18)	−0.014 (3)
N1	0.0333 (12)	0.0280 (11)	0.0354 (12)	−0.0163 (10)	−0.0081 (10)	0.0026 (9)
N2	0.0415 (13)	0.0336 (12)	0.0398 (12)	−0.0271 (11)	−0.0086 (10)	0.0078 (10)
N3	0.0352 (13)	0.0315 (12)	0.0379 (13)	−0.0162 (10)	0.0008 (10)	0.0016 (10)
N4	0.0304 (11)	0.0271 (11)	0.0343 (12)	−0.0093 (10)	0.0023 (9)	−0.0047 (9)
N5	0.0268 (11)	0.0254 (11)	0.0417 (13)	−0.0120 (9)	−0.0010 (10)	−0.0031 (10)
N6	0.0268 (11)	0.0359 (12)	0.0439 (13)	−0.0126 (10)	−0.0095 (10)	−0.0014 (10)
N7A	0.018 (5)	0.027 (6)	0.038 (9)	0.000 (4)	−0.017 (5)	0.018 (5)
N7B	0.066 (5)	0.072 (5)	0.046 (5)	−0.044 (4)	−0.007 (4)	−0.005 (4)
C1	0.0306 (13)	0.0241 (12)	0.0408 (15)	−0.0122 (11)	−0.0054 (11)	0.0041 (11)
C2	0.0374 (14)	0.0297 (13)	0.0412 (15)	−0.0150 (12)	−0.0072 (12)	0.0067 (11)
C3	0.088 (3)	0.081 (3)	0.064 (2)	−0.065 (2)	−0.033 (2)	0.0345 (19)
C4	0.104 (3)	0.103 (3)	0.078 (3)	−0.077 (3)	−0.032 (3)	0.050 (2)
C5	0.078 (3)	0.071 (2)	0.050 (2)	−0.030 (2)	−0.0131 (19)	0.0227 (18)
C6	0.086 (3)	0.074 (2)	0.051 (2)	−0.040 (2)	−0.0280 (19)	0.0132 (18)
C7	0.072 (2)	0.061 (2)	0.0528 (19)	−0.0429 (19)	−0.0210 (17)	0.0143 (16)
C8	0.0278 (13)	0.0377 (14)	0.0286 (13)	−0.0150 (11)	−0.0084 (10)	0.0012 (11)
C9	0.0301 (14)	0.0648 (19)	0.0301 (14)	−0.0244 (14)	−0.0032 (11)	−0.0007 (13)
C10	0.0447 (18)	0.083 (3)	0.051 (2)	−0.0249 (18)	0.0114 (15)	−0.0221 (18)
C11	0.054 (2)	0.134 (4)	0.074 (3)	−0.035 (3)	0.026 (2)	−0.038 (3)
C12	0.060 (3)	0.179 (6)	0.059 (3)	−0.064 (3)	0.017 (2)	−0.014 (3)
C13	0.075 (3)	0.144 (4)	0.059 (2)	−0.080 (3)	−0.007 (2)	0.021 (3)
C14	0.0502 (19)	0.087 (3)	0.0506 (19)	−0.0439 (19)	−0.0074 (15)	0.0128 (18)
C15	0.0331 (14)	0.0297 (13)	0.0428 (15)	−0.0184 (12)	−0.0061 (12)	−0.0020 (11)
C16	0.0355 (14)	0.0465 (16)	0.0438 (16)	−0.0234 (13)	−0.0098 (12)	−0.0026 (13)
C17	0.057 (2)	0.064 (2)	0.055 (2)	−0.0227 (17)	−0.0228 (17)	−0.0007 (17)
C18	0.078 (3)	0.099 (3)	0.059 (2)	−0.033 (2)	−0.035 (2)	0.008 (2)
C19	0.076 (3)	0.120 (4)	0.051 (2)	−0.053 (3)	−0.017 (2)	−0.012 (2)
C20	0.073 (2)	0.078 (3)	0.056 (2)	−0.041 (2)	0.0006 (19)	−0.0246 (19)
C21	0.0538 (19)	0.0540 (19)	0.0530 (19)	−0.0261 (16)	−0.0056 (15)	−0.0091 (15)
C22	0.0292 (13)	0.0189 (11)	0.0365 (14)	−0.0116 (10)	−0.0051 (11)	−0.0014 (10)
C23	0.0227 (11)	0.0205 (11)	0.0303 (12)	−0.0105 (9)	−0.0044 (10)	0.0005 (9)
C24	0.0215 (11)	0.0209 (11)	0.0283 (12)	−0.0106 (9)	−0.0039 (9)	−0.0006 (9)
C25	0.0238 (11)	0.0197 (11)	0.0314 (12)	−0.0117 (9)	−0.0075 (10)	0.0002 (9)
C26	0.0268 (12)	0.0204 (11)	0.0382 (14)	−0.0123 (10)	−0.0081 (11)	−0.0005 (10)
C27	0.0320 (13)	0.0282 (13)	0.0448 (15)	−0.0201 (11)	0.0016 (11)	−0.0016 (11)
C28	0.0286 (13)	0.0324 (14)	0.0534 (17)	−0.0154 (11)	0.0117 (12)	−0.0102 (12)
C29	0.0290 (13)	0.0210 (12)	0.0448 (15)	−0.0099 (10)	0.0032 (11)	−0.0087 (11)

### Geometric parameters (Å, º)

**Table:**

Sm1—O1	2.4415 (17)	C3—C4	1.385 (5)
Sm1—O2	2.3973 (17)	C4—H4A	0.9300
Sm1—O3	2.4775 (17)	C4—C5	1.348 (5)
Sm1—O4	2.4024 (16)	C5—H5	0.9300
Sm1—O8	2.3397 (19)	C5—C6	1.344 (5)
Sm1—O9	2.4827 (18)	C6—H6A	0.9300
Sm1—N1	2.694 (2)	C6—C7	1.375 (4)
Sm1—N3	2.680 (2)	C7—H7	0.9300
Sm1—N5	2.633 (2)	C8—C9	1.487 (3)
O1—C1	1.243 (3)	C9—C10	1.381 (4)
O2—C8	1.240 (3)	C9—C14	1.380 (4)
O3—C15	1.246 (3)	C10—H10	0.9300
O4—C22	1.277 (3)	C10—C11	1.384 (5)
O5—C22	1.238 (3)	C11—H11	0.9300
O6—C26	1.262 (3)	C11—C12	1.346 (7)
O7—C26	1.244 (3)	C12—H12	0.9300
O8—H8A	0.834 (10)	C12—C13	1.358 (7)
O8—H8B	0.837 (10)	C13—H13	0.9300
O9—H9A	0.825 (10)	C13—C14	1.395 (5)
O9—H9B	0.831 (10)	C14—H14	0.9300
O10A—N7A	1.238 (12)	C15—C16	1.490 (4)
O10B—N7B	1.234 (8)	C16—C17	1.383 (4)
O11A—N7A	1.242 (13)	C16—C21	1.380 (4)
O11B—N7B	1.235 (8)	C17—H17	0.9300
O12A—N7A	1.257 (12)	C17—C18	1.384 (5)
O12B—N7B	1.238 (8)	C18—H18	0.9300
N1—H1A	0.875 (10)	C18—C19	1.368 (6)
N1—H1B	0.878 (10)	C19—H19	0.9300
N1—N2	1.413 (3)	C19—C20	1.356 (5)
N2—H2	0.871 (10)	C20—H20	0.9300
N2—C1	1.320 (3)	C20—C21	1.387 (5)
N3—H3A	0.870 (10)	C21—H21	0.9300
N3—H3B	0.878 (10)	C22—C23	1.492 (3)
N3—N4	1.410 (3)	C23—C24	1.384 (3)
N4—H4	0.867 (10)	C23—C29	1.387 (3)
N4—C8	1.322 (3)	C24—H24	0.9300
N5—H5A	0.879 (10)	C24—C25	1.388 (3)
N5—H5B	0.871 (10)	C25—C26	1.501 (3)
N5—N6	1.410 (3)	C25—C27	1.385 (3)
N6—H6	0.873 (10)	C27—H27	0.9300
N6—C15	1.319 (3)	C27—C28	1.379 (3)
C1—C2	1.489 (3)	C28—H28	0.9300
C2—C3	1.373 (4)	C28—C29	1.381 (3)
C2—C7	1.370 (4)	C29—H29	0.9300
C3—H3	0.9300		
			
O1—Sm1—O3	72.08 (6)	C7—C2—C1	117.9 (2)
O1—Sm1—O9	133.85 (6)	C7—C2—C3	118.2 (3)
O1—Sm1—N1	60.97 (6)	C2—C3—H3	120.1
O1—Sm1—N3	125.95 (7)	C2—C3—C4	119.8 (3)
O1—Sm1—N5	67.85 (6)	C4—C3—H3	120.1
O2—Sm1—O1	77.69 (6)	C3—C4—H4A	119.5
O2—Sm1—O3	79.96 (6)	C5—C4—C3	121.0 (3)
O2—Sm1—O4	131.15 (6)	C5—C4—H4A	119.5
O2—Sm1—O9	80.28 (6)	C4—C5—H5	120.2
O2—Sm1—N1	68.37 (6)	C6—C5—C4	119.6 (3)
O2—Sm1—N3	61.71 (6)	C6—C5—H5	120.2
O2—Sm1—N5	134.49 (7)	C5—C6—H6A	119.8
O3—Sm1—O9	141.80 (6)	C5—C6—C7	120.5 (3)
O3—Sm1—N1	127.30 (6)	C7—C6—H6A	119.8
O3—Sm1—N3	67.24 (7)	C2—C7—C6	120.9 (3)
O3—Sm1—N5	62.03 (6)	C2—C7—H7	119.5
O4—Sm1—O1	144.08 (6)	C6—C7—H7	119.5
O4—Sm1—O3	90.56 (6)	O2—C8—N4	121.2 (2)
O4—Sm1—O9	78.51 (6)	O2—C8—C9	120.1 (2)
O4—Sm1—N1	141.91 (6)	N4—C8—C9	118.7 (2)
O4—Sm1—N3	70.39 (6)	C10—C9—C8	122.9 (3)
O4—Sm1—N5	76.25 (6)	C14—C9—C8	117.6 (3)
O8—Sm1—O1	95.65 (7)	C14—C9—C10	119.5 (3)
O8—Sm1—O2	137.16 (6)	C9—C10—H10	120.0
O8—Sm1—O3	138.72 (7)	C9—C10—C11	120.1 (4)
O8—Sm1—O4	76.66 (6)	C11—C10—H10	120.0
O8—Sm1—O9	74.43 (7)	C10—C11—H11	119.9
O8—Sm1—N1	71.49 (7)	C12—C11—C10	120.2 (4)
O8—Sm1—N3	138.35 (7)	C12—C11—H11	119.9
O8—Sm1—N5	76.77 (7)	C11—C12—H12	119.6
O9—Sm1—N1	73.31 (6)	C11—C12—C13	120.8 (4)
O9—Sm1—N3	74.63 (7)	C13—C12—H12	119.6
O9—Sm1—N5	145.24 (6)	C12—C13—H13	119.8
N3—Sm1—N1	123.90 (7)	C12—C13—C14	120.4 (4)
N5—Sm1—N1	115.01 (7)	C14—C13—H13	119.8
N5—Sm1—N3	117.67 (7)	C9—C14—C13	119.0 (4)
C1—O1—Sm1	124.68 (16)	C9—C14—H14	120.5
C8—O2—Sm1	126.68 (16)	C13—C14—H14	120.5
C15—O3—Sm1	120.32 (16)	O3—C15—N6	121.8 (2)
C22—O4—Sm1	130.94 (14)	O3—C15—C16	120.6 (2)
Sm1—O8—H8A	144 (2)	N6—C15—C16	117.5 (2)
Sm1—O8—H8B	104 (3)	C17—C16—C15	121.6 (3)
H8A—O8—H8B	109 (3)	C21—C16—C15	119.1 (3)
Sm1—O9—H9A	110.4 (19)	C21—C16—C17	119.2 (3)
Sm1—O9—H9B	117 (2)	C16—C17—H17	120.0
H9A—O9—H9B	106 (3)	C16—C17—C18	120.1 (3)
Sm1—N1—H1A	114.3 (18)	C18—C17—H17	120.0
Sm1—N1—H1B	109.2 (19)	C17—C18—H18	119.9
H1A—N1—H1B	105 (3)	C19—C18—C17	120.2 (4)
N2—N1—Sm1	111.02 (14)	C19—C18—H18	119.9
N2—N1—H1A	107.0 (17)	C18—C19—H19	120.0
N2—N1—H1B	110.1 (19)	C20—C19—C18	120.0 (4)
N1—N2—H2	119.1 (18)	C20—C19—H19	120.0
C1—N2—N1	117.4 (2)	C19—C20—H20	119.6
C1—N2—H2	123.5 (18)	C19—C20—C21	120.7 (4)
Sm1—N3—H3A	106 (2)	C21—C20—H20	119.6
Sm1—N3—H3B	116 (2)	C16—C21—C20	119.7 (3)
H3A—N3—H3B	113 (3)	C16—C21—H21	120.1
N4—N3—Sm1	111.54 (14)	C20—C21—H21	120.1
N4—N3—H3A	107 (2)	O4—C22—C23	118.50 (19)
N4—N3—H3B	103 (2)	O5—C22—O4	123.6 (2)
N3—N4—H4	116 (2)	O5—C22—C23	117.9 (2)
C8—N4—N3	117.1 (2)	C24—C23—C22	120.8 (2)
C8—N4—H4	126 (2)	C24—C23—C29	119.3 (2)
Sm1—N5—H5A	106.2 (18)	C29—C23—C22	119.9 (2)
Sm1—N5—H5B	113.6 (18)	C23—C24—H24	119.5
H5A—N5—H5B	110 (2)	C23—C24—C25	120.9 (2)
N6—N5—Sm1	111.50 (14)	C25—C24—H24	119.5
N6—N5—H5A	107.1 (18)	C24—C25—C26	121.1 (2)
N6—N5—H5B	108.7 (18)	C27—C25—C24	118.8 (2)
N5—N6—H6	119.2 (19)	C27—C25—C26	120.1 (2)
C15—N6—N5	117.6 (2)	O6—C26—C25	119.4 (2)
C15—N6—H6	123.1 (19)	O7—C26—O6	123.5 (2)
O10A—N7A—O11A	116.6 (12)	O7—C26—C25	117.1 (2)
O10A—N7A—O12A	124.6 (13)	C25—C27—H27	119.6
O11A—N7A—O12A	118.5 (13)	C28—C27—C25	120.9 (2)
O10B—N7B—O11B	118.4 (7)	C28—C27—H27	119.6
O10B—N7B—O12B	119.9 (7)	C27—C28—H28	120.1
O11B—N7B—O12B	121.7 (7)	C27—C28—C29	119.8 (2)
O1—C1—N2	120.8 (2)	C29—C28—H28	120.1
O1—C1—C2	120.2 (2)	C23—C29—H29	119.9
N2—C1—C2	119.0 (2)	C28—C29—C23	120.3 (2)
C3—C2—C1	123.9 (3)	C28—C29—H29	119.9
			
Sm1—O1—C1—N2	20.2 (3)	C3—C2—C7—C6	0.4 (5)
Sm1—O1—C1—C2	−159.95 (17)	C3—C4—C5—C6	0.0 (7)
Sm1—O2—C8—N4	8.8 (3)	C4—C5—C6—C7	0.2 (6)
Sm1—O2—C8—C9	−171.58 (16)	C5—C6—C7—C2	−0.4 (6)
Sm1—O3—C15—N6	24.0 (3)	C7—C2—C3—C4	−0.3 (5)
Sm1—O3—C15—C16	−155.18 (18)	C8—C9—C10—C11	178.8 (3)
Sm1—O4—C22—O5	17.8 (4)	C8—C9—C14—C13	−179.2 (3)
Sm1—O4—C22—C23	−161.23 (15)	C9—C10—C11—C12	0.1 (6)
Sm1—N1—N2—C1	−16.1 (3)	C10—C9—C14—C13	−1.1 (5)
Sm1—N3—N4—C8	−12.0 (3)	C10—C11—C12—C13	−0.7 (7)
Sm1—N5—N6—C15	−17.1 (3)	C11—C12—C13—C14	0.4 (7)
O1—C1—C2—C3	−176.1 (3)	C12—C13—C14—C9	0.5 (6)
O1—C1—C2—C7	5.1 (4)	C14—C9—C10—C11	0.8 (5)
O2—C8—C9—C10	−179.7 (3)	C15—C16—C17—C18	−176.6 (3)
O2—C8—C9—C14	−1.7 (4)	C15—C16—C21—C20	178.2 (3)
O3—C15—C16—C17	146.7 (3)	C16—C17—C18—C19	−1.1 (6)
O3—C15—C16—C21	−29.8 (4)	C17—C16—C21—C20	1.6 (5)
O4—C22—C23—C24	0.4 (3)	C17—C18—C19—C20	0.7 (6)
O4—C22—C23—C29	179.4 (2)	C18—C19—C20—C21	0.9 (6)
O5—C22—C23—C24	−178.7 (2)	C19—C20—C21—C16	−2.0 (5)
O5—C22—C23—C29	0.4 (4)	C21—C16—C17—C18	−0.1 (5)
N1—N2—C1—O1	0.1 (4)	C22—C23—C24—C25	179.5 (2)
N1—N2—C1—C2	−179.8 (2)	C22—C23—C29—C28	−179.2 (2)
N2—C1—C2—C3	3.8 (4)	C23—C24—C25—C26	−179.3 (2)
N2—C1—C2—C7	−175.0 (3)	C23—C24—C25—C27	−0.6 (3)
N3—N4—C8—O2	4.0 (4)	C24—C23—C29—C28	−0.1 (4)
N3—N4—C8—C9	−175.7 (2)	C24—C25—C26—O6	−9.3 (3)
N4—C8—C9—C10	0.0 (4)	C24—C25—C26—O7	170.6 (2)
N4—C8—C9—C14	178.0 (2)	C24—C25—C27—C28	0.5 (4)
N5—N6—C15—O3	−2.7 (4)	C25—C27—C28—C29	−0.2 (4)
N5—N6—C15—C16	176.5 (2)	C26—C25—C27—C28	179.2 (2)
N6—C15—C16—C17	−32.5 (4)	C27—C25—C26—O6	172.0 (2)
N6—C15—C16—C21	151.0 (3)	C27—C25—C26—O7	−8.1 (4)
C1—C2—C3—C4	−179.0 (3)	C27—C28—C29—C23	0.0 (4)
C1—C2—C7—C6	179.3 (3)	C29—C23—C24—C25	0.4 (3)
C2—C3—C4—C5	0.1 (7)		

### Hydrogen-bond geometry (Å, º)

Cg1 and Cg3 are the centroids of the C2–C7 and C16–C21 rings, respectively.

**Table:**

*D*—H···*A*	*D*—H	H···*A*	*D*···*A*	*D*—H···*A*
O8—H8*A*···O7^i^	0.83 (2)	1.75 (2)	2.574 (2)	168 (3)
O8—H8*B*···O5	0.84 (2)	1.76 (2)	2.585 (3)	166 (3)
O9—H9*A*···O12*A*^ii^	0.83 (2)	2.22 (2)	2.988 (2)	156 (3)
O9—H9*A*···O12*B*^ii^	0.83 (2)	2.09 (2)	2.888 (2)	162 (3)
O9—H9*B*···O6^ii^	0.83 (2)	1.81 (2)	2.633 (2)	170 (3)
N1—H1*A*···O6^i^	0.88 (2)	2.14 (2)	3.012 (3)	172 (2)
N1—H1*B*···O6^ii^	0.88 (2)	2.14 (2)	2.965 (3)	156 (3)
N2—H2···O11*A*^i^	0.87 (2)	2.22 (3)	2.936 (2)	140 (2)
N2—H2···O12*B*^i^	0.87 (2)	2.10 (2)	2.946 (3)	164 (3)
N3—H3*B*···O4^ii^	0.88 (2)	2.53 (2)	3.344 (3)	155 (3)
N4—H4···O10*B*	0.87 (2)	2.18 (2)	3.040 (3)	176 (3)
N4—H4···O12*A*	0.87 (2)	2.31 (3)	2.970 (2)	133 (3)
N5—H5*A*···O5^iii^	0.88 (2)	2.07 (2)	2.878 (3)	152 (2)
N5—H5*B*···O5	0.87 (2)	2.67 (2)	3.199 (3)	120 (2)
N5—H5*B*···O7^iv^	0.87 (2)	2.15 (2)	2.940 (3)	151 (2)
N6—H6···O10*A*^v^	0.87 (2)	2.22 (3)	2.961 (2)	142 (2)
N6—H6···O11*B*^v^	0.87 (2)	2.28 (2)	3.115 (3)	159 (3)
C10—H10···O10*B*	0.93	2.36	3.282 (2)	173
C11—H11···*Cg*1^vi^	0.93	3.10	3.866 (2)	137
C13—H13···*Cg*3^vii^	0.93	3.02	3.712 (4)	132

Symmetry codes: (i) *x*, *y*−1, *z*; (ii) −*x*+1, −*y*+1, −*z*+1; (iii) −*x*+2, −*y*, −*z*+1; (iv) −*x*+2, −*y*+1, −*z*+1; (v) *x*+1, *y*−1, *z*; (vi) *x*−1, *y*+1, *z*; (vii) *x*−1, *y*, *z*.
